# Tunable network properties with Hamill and Gilbert’s Social Circles generator

**DOI:** 10.1007/s41109-025-00744-5

**Published:** 2025-12-04

**Authors:** Cristina Chueca Del Cerro, Jennifer Badham

**Affiliations:** Department of Sociology, Durham University, Mill Hill Lane, Durham, DH1 3LB UK

**Keywords:** Social network properties, Network generator, Network property relationships

## Abstract

Hamill and Gilbert (J Artif Soc Soc Simul 12, 1–23, 2009) developed the Social Circles algorithm to generate synthetic networks that have properties of real social networks such as skewed degree distribution, positive clustering coefficient, degree assortativity and short path lengths. To assess the viability of Social Circles as a general network generator, we systematically examine the relationship between algorithm parameters and a broader range of structural properties of the generated networks. We varied social reaches for agents, distribution of social reaches in the population, and node density. We find that edge density and centrality measures can be controlled in a predictable way: longer reaches are associated with denser networks, shorter paths, lower degree assortativity (with some exceptions), and smaller variation in centrality measures. However, these network properties changed together and there is limited capacity to control properties separately. Further, clustering coefficient is insensitive to algorithm inputs. Thus, it cannot be used as a general network generator as it stands. If these properties are important, Social Circles could be used to generate starting networks with reasonable social structure, but further steps would be required to refine the structural properties.

## Introduction

Simulations have been widely used to explore the dynamics of processes that occur over social networks, such as epidemic spread (Almagor and Picascia 2020) or behaviour adoption (Valente and Vega Yon 2020). The influence of the network structure can be investigated by applying the same simulation rules over networks that differ with respect to the specific structural properties of interest (Badham and Stocker 2010). For social dynamics, such studies are only valid to the extent that the networks are similar to real world social networks.

The structure of social networks differs from that of other types of networks (Newman and Park 2003) and also from the widely used simplistic generators of synthetic networks that are available in network software (Hamill and Gilbert 2009). In response, researchers have published a wide variety of algorithms to generate synthetic networks with more realistic structural properties (Staudt et al 2017; Gursoy and Badur 2019).

Hamill and Gilbert (2009) present one such generator based on understanding of a personal network as a set of intersecting social circles (Simmel 1902). In the Social Circles (SC) network generator, agents (nodes in the network to be generated) are located in a notional space and each assigned a social reach (*sr*). Agents form connections with other agents within their social reach who can also reciprocate the connection. That is, pairs of agents form network edges when the shorter reach of the pair is at least the distance between the pair.

The distribution of *sr* among agents controls the properties of the generated networks. The objective of the SC algorithm (Hamill and Gilbert 2009) was to create networks with properties that are similar to those of real world social networks: low density, limited degree, variation in degree, high clustering coefficient and assortativity, and the presence of communities.

Our objective is to explore the potential of SC as a general network generator of more realistic networks. That is, can networks be generated that have specific combinations of properties by manipulating the input parameters of the algorithm? We extend the analysis of the SC model in two ways. We measure a more complete range of network properties and more systematically vary the distribution of social reach values for two specific variants of the model.

The paper is organised as follows. We describe our implementation of the Social Circles algorithm and the experimental design of social reach distributions. Some of the distributions are identical to those in the original paper to ensure we have implemented the algorithm correctly. This is followed by results in two broad groups; the relationship between algorithm parameters and network properties, and the relationship between different sets of network properties. The discussion focuses on the implications of our findings for the potential use of the SC model as a flexible network generator.

## Methodology

The published model by Hamill and Gilbert (2009) was implemented with NetLogo (Wilensky 1999). We re-implemented the algorithm in Julia (Bezanson et al 2017) and generated networks with an extended set of parameter combinations. This section explains our experimental design and measurement of structural properties. All the analyses were conducted in R (R Core Team 2024). All source code and data can be found at OSF repository. More details are provided in the Availability of data and materials section.

### Social circles, as published

The first step of the Social Circles algorithm (Hamill and Gilbert 2009) is to randomly locate 1000 agents (which become nodes in the network) across the 99,225 grid positions of a notional wrapped 315x315 torus. That is, approximately 1% of the available positions are occupied. The nodes are assigned a social reach (*sr*) according to some distribution. In the final step, each pair of nodes forms an edge if each node in the pair is within the social reach of the other. An appropriate scale for reaches can be estimated from the maximum distance between a pair of nodes of 222 (calculated as: $\sqrt{2 * 157 ^ 2}$).

The simplest model (referred to as ‘One Circle’ in the original paper) assigned the same social reach to each node. Four values of *sr* were tested: 15, 30, 40, and 50 (Section 3.6 in original paper). The average degree is directly related to the social reach; a larger reach results in a denser network. Overall, smaller values of *sr* led to disconnected networks and larger values to more connected ones.

In order to explore further the effects of social reaches, Hamill and Gilbert (2009) extended the model to have two (‘Two Circles’) and three (‘Three Circles’) unique social reaches. They tested different combinations of social reach values and proportions of agents allocated each value. Additionally, Hamill and Gilbert (2009) tested two other configurations that allowed agents to take any value from continuous social reach distributions: Poisson with mean 30, and uniform over the range 10 to 50 (Section 4.9 in original paper).

### Experimental design

Our experimental design focuses on these models that select from specific *sr* values and different distributions in the population. These involve two parameters: the specific social reach values available (two or three values), and the proportion of nodes allocated each value. We varied these two parameters separately and in combination.

Eight combinations of parameter values are examined. There are five combinations with two reach values. These provide two sets of three experiments that systematically vary one parameter (values or distribution) across the set, with one combination occurring in both sets. This structure allows us to examine each set of three experiments for consistent effects on network properties. The other three experiments fix three values of social reach and vary only the distribution, to consider the effect of an additional potential value for social reach. The parameter combinations are shown in Table 1.

**Table 1:** Experimental design: values for social reaches and the proportion of nodes with the shortest (SR), medium (MR, only if 3 reach values) and longest (LR) social reach.

Experiment^1^	Reach Values	Prop SR	Prop MR	Prop LR
Two *sr* values: Vary values, fixed proportions				
Original	15 and 30	75	-	25
Additional^2^	20 and 40	75	-	25
Original	30 and 50	75	-	25
Two *sr* values: Fixed values, vary proportions				
Additional	20 and 40	90	-	10
Additional	20 and 40	75	-	25
Additional	20 and 40	25	-	75
Three *sr* values: Fixed values, vary proportions				
Additional	30, 40 and 50	10	20	70
Original	30, 40 and 50	34	33	33
Original	30, 40 and 50	70	20	10

^1^Identifies whether the parameter values were included in the original paper (Hamill and Gilbert 2009).

^2^This experiment is listed twice in the table for readability.

Four of these experiments serve an additional purpose of verifying that we have accurately replicated the original algorithm. Hamill and Gilbert (2009) reported results from two ‘Two Circle’ (two reach values) and two ‘Three Circle’ (three reach values) experiments. These values and distributions are included in our experimental design, allowing us to compare the properties of generated networks between the two implementations. These experiments were all run with 1,000 nodes, to be consistent with the original design.

We generated 30 networks for each combination of social reach value and distribution parameters in Table 1. For each network, we measured the properties on the giant component, summarised in Table 2. These cover both average and variation for common centrality measures, clustering coefficients and geodesics (shortest path lengths). Note that we use the Gini coefficient to measure variation to better deal with non-normal distributions (Badham 2013). Several of those properties were also measured by Hamill and Gilbert (2009), which allows verification of our implementation.

**Table 2:** Measure structural network properties for the Social Circles and our model.

Measured Property	Social	Our	Comment
	Circles	Analysis	
Density	$\checkmark$	$\checkmark$	
Number of edges	$\checkmark$	$\checkmark$	
Degree mean	$\checkmark$	$\checkmark$	
Degree variation	$\checkmark$	$\checkmark$	Different measures
Assortativity	$\checkmark$	$\checkmark$	Different measures
Closeness mean		$\checkmark$	
Closeness variation		$\checkmark$	
Betweenness mean		$\checkmark$	
Betweenness variation		$\checkmark$	
Eigenvector mean		$\checkmark$	
Eigenvector variation		$\checkmark$	
Clustering coefficient mean	$\checkmark$	$\checkmark$	
Clustering coefficient variation	$\checkmark$	$\checkmark$	Different measures
Global transitivity		$\checkmark$	
Geodesic mean		$\checkmark$	
Geodesic variation		$\checkmark$	
Diameter		$\checkmark$	

Separately, we repeated all the experiments in Table 1 but with 2,000 nodes to explore whether changing the node density would allow different manipulations of these properties. Increasing the density is equivalent to increasing the reach of all nodes at lower density as it increases the number of nodes reached in the same distance. The relationships between parameters and properties were the same as for the 1,000 node experiments, hence they are not reported.

## Results

We first present the properties of the networks generated with the four parameter combinations that appear in both our analysis and that of Hamill and Gilbert (2009) to verify our successful re-implementation. We then compare the degree distributions for nodes with different social reaches in each set of experiments, to help interpret the results as reach distributions vary.

The next three sets of results examine the relationship between social reach values, the distribution of those reaches, and network properties. Finally, we analyse the relationship between network properties, to assess the capacity of the SC algorithm to target specific property values of interest.

### Verifying the Social Circles implementation

From Table 3, it is clear that the 1,000 node networks generated with our implementation of the Social Circles algorithm have very similar edge counts as those generated by Hamill and Gilbert (2009, Figures 9 and 11). Where average clustering coefficient was reported, these values are also consistent between implementations. Average assortativity values are clearly different, but these are different calculations. Hamill and Gilbert (2009, paragraph 3.11) calculate the correlation between a node’s degree and the average degree of its neighbours. In contrast, we reported correlation coefficient between degrees of the pairs of nodes at the ends of each edge (Newman 2002).

**Table 3:** Properties^1^ of networks generated by original (HG) and re-implemented (New) Social Circles algorithm, average over 30 networks.

Distribution	Social reaches	Mean degree		Clustering coeff		Assortativity	
		HG	New	HG	New	HG	New
Two circles^1^							
75%, 25%	[15,30]	8	8	0.54	0.559	0.71	0.429
75%, 25%	[30,50]	32	32	0.56	0.573	0.75	0.364
Three circles^2^							
70%, 20%, 10%	[30,40,50]	31	31		0.580		0.416
34%, 33%, 33%	[30,40,50]	41	41		0.591		0.267

^1^The values in parenthesis indicate the proportion of short-reach (left) and long-reach (right) nodes.

^2^The values in parenthesis indicate the proportion of short-reach, medium-reach and long-reach nodes, from left to right.

We also successfully matched the mean degree and clustering coefficient reported by Hamill and Gilbert (2009) for ‘One Circle’ experiments with social reach of 30 and for continuous reaches drawn from Poisson distribution (not reported here, but available from the authors). However, our networks using reaches drawn from a random uniform distribution consistently generated too few edges. This was not investigated further as it likely reflects use of integer distances and our interest is the influence of the adjustable parameters of specific social reaches and their distribution reflected in the Two and Three Circle experiments.

### Degree distributions for specific values of social reaches

Social reach governs the opportunities for a node to create edges. A longer reach means there are more nodes within the social circle (on average) and therefore more potential edges for that node. Intuitively therefore we expect longer reaches to be associated with higher degrees and that is what was found by Hamill and Gilbert (2009). We explore this relationship by comparing the degree of nodes with different values of social reach within the same networks.

**Fig. 1:**
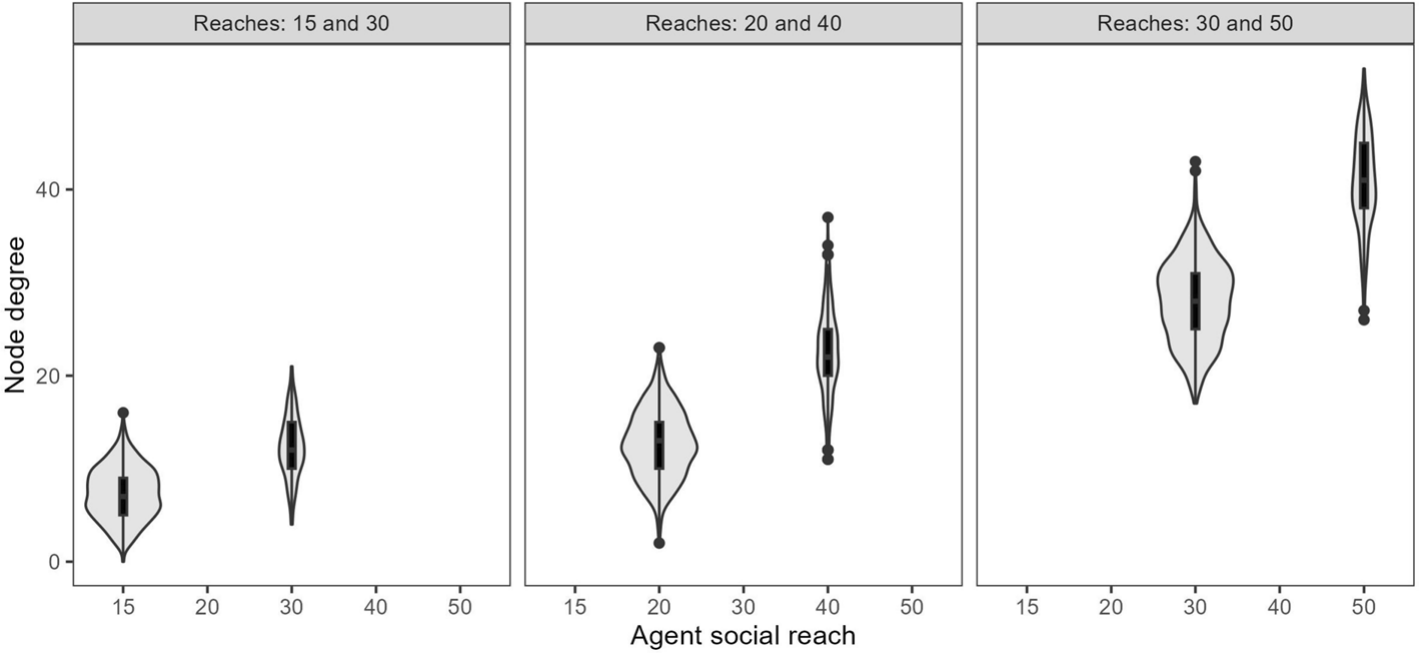
Distribution of node degrees by social reach with varied reach values and fixed proportions of 75% short-reach and 25% long-reach nodes. Longer reaches tend to be associated with higher degree.

In Fig. 1, the proportion of short-reach nodes is fixed at 75% and longer reach at 25% but the values of reach increase from left to right. In each panel, the degree of the longer reach nodes is generally higher than the degree for the shorter reach nodes, as expected. However, there is overlap between the degree distributions for each set of networks. In the networks with reaches of 15 and 30 (left panel), the lowest degree nodes with reach 30 have lower degrees than the highest degree nodes with reach 15, and similarly for the other pairs of reach values. This variety of degree values and overlap of degree distributions indicates that reach does not fully determine degree.

**Fig. 2:**
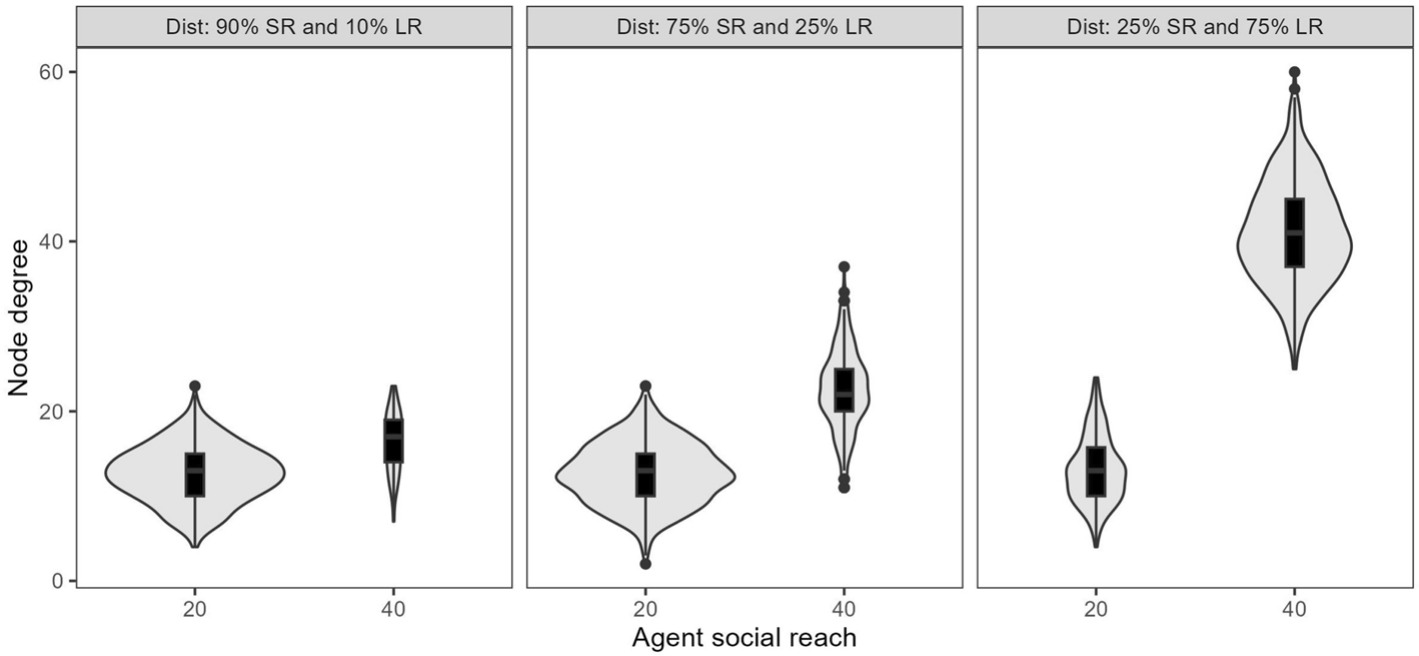
Distribution of node degrees by social reach with fixed reach values and varied proportions of 75% short-reach and 25% long-reach nodes. Higher proportions of long-reach nodes are associated with higher degrees for the long-reach nodes but no change for the short-reach nodes.

Longer reach nodes also have higher degree in those experiments where the values of short- and long-reach are constant but the proportions of each type of node vary (Fig. 2). Further, the degree distribution for the shorter reach nodes (social reach of 20) is similar regardless of the proportions. In contrast, the longer reach nodes have much higher degrees where the proportion of these nodes is high. That is, the overlap between the degree distributions of the shorter and longer reach nodes varies as the proportions change. This is a consequence of the requirement for reciprocity. With a higher proportion of longer reach nodes, the opportunities to create an edge arising from that longer reach can be converted to actual edges because the reached node is more likely to also have a long social reach.

The same broad patterns were observed in the experiments with 2,000 agents. Overall, the degrees are higher as expected due to the larger number of agents, but the same relationships exist between the degree distributions of the shorter and longer reach nodes (results in the Appendix).

### Varying social reach: Two social reaches

Our first set of experiments varied the social reaches assigned to nodes while fixing the population composition at 75% short-reach and 25% long-reach nodes. As expected from the relationship between degree and reach, longer reach values are associated with more edges in the networks (Table 4). The edges increase faster than the reaches; the mean degree for the networks with the longest reach values was more than three times the mean degree of those with the shortest reach values despite the reach values only (up to) doubling. The increase in average degree is associated with a moderate decrease in degree variability, with Gini of degree falling from 0.243 to 0.137.

**Table 4:** Varying social reach values (2 values): General network properties.

Composition	Reaches	Edges	Density	Degree		Assortativity
SR, LR	[SR,LR]			Mean	Gini	
75%, 25%	[15,30]	4,261	0.009	8.5	0.243	0.429
75%, 25%	[20,40]	7,498	0.015	15.0	0.204	0.390
75%, 25%	[30,50]	15,787	0.032	31.6	0.137	0.364

Focusing on other centrality measures, closeness and eigenvector centrality show the same pattern as degree, with an increase in mean and decrease in Gini coefficient as the reach values increase (Table 5). The reduction in Gini coefficient of eigenvector centrality is substantial but it starts from the extremely high value of 0.942. From a visual inspection of some example networks, communities form around small sets of relatively close long-reach nodes. While the bulk of the network is well distributed, a small number of nodes have high eigenvector centrality values because they sit within these communities. Where there is a higher proportion of long-reach nodes, the nodes are more evenly distributed throughout the network.

**Table 5:** Varying social reach values (2 values): Centrality measures.

Composition	Reaches	Closeness		Betweenness		Eigenvector	
SR, LR	[SR,LR]	Mean	Gini	Mean	Gini	Mean	Gini
75%, 25%	[15,30]	0.129	0.043	0.007	0.758	0.007	0.942
75%, 25%	[20,40]	0.189	0.039	0.004	0.761	0.012	0.819
75%, 25%	[30,50]	0.250	0.032	0.003	0.695	0.019	0.597

These communities also affect betweenness values, which measures the extent to which a node lies on shortest paths between pairs of nodes. Average betweenness centrality is close to zero for all networks (Table 5). Very low values indicates that the networks are generally very well connected, all nodes are easily reached from each other. However, Gini coefficient of betweenness is high, which means there are a small number of nodes bridging these communities. Gini of betweenness is lower with higher proportions of longer reach nodes, indicating that these networks are more strongly connected.

Assortativity is fairly high for all networks but it decreases as social reaches increase (Table 4). However, the effect is small, decreasing from 0.429 to 0.364 (15% decline) despite almost doubling the reach values.

Focusing now on triads in the networks (Table 6), average local clustering coefficient is high across all networks with only minor differences across experimental conditions. In contrast, Gini coefficient was moderate and decreased substantially, indicating that the variation in the level of cohesion around nodes reduces as the social reaches increases. Transitivity is also high and insensitive to changes in reach values.

**Table 6:** Varying social reach values (2 values) : Clustering and Geodesics.

Composition	Reaches	Clustering Coeff		Transitivity	Geodesic		Diameter
SR, LR	[SR,LR]	Mean	Gini		Mean	Gini	
75%, 25%	[15,30]	0.562	0.182	0.514	7.820	0.187	16
75%, 25%	[20,40]	0.562	0.126	0.513	5.323	0.173	8
75%, 25%	[30,50]	0.572	0.082	0.549	4.017	0.171	7

Lastly, we were interested in distances between nodes within the network (Table 6), which show a substantial affected with changes in the social reaches. The increased edge density (see Table 4) as social reaches increase is associated with a decrease in distances between nodes. Both the average geodesic and diameter (maximum geodesic) consistently fall, with a particularly large drop in diameter as reaches change from 15 and 30 to 20 and 40 and a much smaller change for the longest reaches. Variation in geodesics between pairs of nodes also decreases, suggesting that the additional edges as social reaches increase are facilitating connectivity between all pairs of nodes.

### Varying reach proportions: Two social reaches

Our second set of experiments kept the social reaches available for nodes constant (at 20 and 40) but varied the mix of these reaches in the node population. There were more edges in the network as the proportion of longer reach agents increased, reflected also as higher values of mean degree and density (Table 7). That is, the nodes within the reach of longer reach nodes are more likely to themselves be longer reach and able to reciprocate to create an edge. This pattern is consistent with our previous results that longer reaches are associated with more edges; intuitively, increasing the average reach of nodes can be achieved by either increasing the proportion of longer reaches or increasing reach values.

**Table 7:** Varying social reach proportions (2 values): General network properties.

Composition	Reaches	Edges	Density	Degree		Assortativity
SR, LR	[SR,LR]			Mean	Gini	
90%, 10%	[20,40]	6,516	0.013	13.0	0.162	0.520
75%, 25%	[20,40]	7,498	0.015	15.0	0.204	0.390
25%, 75%	[20,40]	17,028	0.034	34.1	0.219	0.295

In contrast to the experiments with increased reach values, the increase in edges as the proportion of longer reach nodes increased was associated with an increase in the variation (Gini coefficient) in degree from 0.162 to 0.219. This pattern can also be seen in Fig. 2, where higher proportions of long-reach nodes separated the degree distributions of the short and long-reach nodes.

We found that increasing the proportion of long-reach agents increased the average closeness and eigenvector centralities (Table 8). This pattern is consistent with the other set of experiments where more edges was associated with higher closeness and eigenvector centralities (Table 5). Variation in eigenvector centrality substantially decreases from 0.927 to 0.594, which is also consistent with the previous patterns. However, there is no pattern for the Gini coefficient of closeness centrality.

**Table 8:** Varying social reach proportions (2 values): Centrality measures.

Composition	Reaches	Closeness		Betweenness		Eigenvector	
SR, LR	[SR,LR]	Mean	Gini	Mean	Gini	Mean	Gini
90%, 10%	[20,40]	0.148	0.032	0.006	0.677	0.008	0.927
75%, 25%	[20,40]	0.189	0.039	0.004	0.761	0.012	0.819
25%, 75%	[20,40]	0.244	0.031	0.003	0.421	0.019	0.594

Once more we found that the average betweenness centrality was close to zero across all experiments (Table 8). This was accompanied by a very high Gini coefficient that decreased sharply as long-reach agents became the majority in the population. That is, communities within the network are less concentrated for the experiments with more long-reach agents.

Assortativity decreased substantially, from 0.520 to 0.295, as the proportion of long-reach agents in the population increased (see Table 7). This relationship again reflects the separation of the degree distributions displayed in Figure 2 and the increased Gini coefficient of degree (Table 7). With a much wider range of node degrees generated in the networks with a high proportion of long-reach nodes, it is more likely that edges will connect pairs of nodes with very different degrees.

There is no pattern in mean or variation of clustering coefficient as the population composition changed (Table 9) with small changes in both directions. Mean local clustering coefficient and transitivity are consistently high at over 0.5, with a large proportion of triangles in the network regardless of the mix of reaches. Gini of clustering coefficient is low across all experiments.

**Table 9:** Varying social reach proportions (2 values): Clustering and Geodesics.

Composition	Reaches	Clustering Coeff		Transitivity	Geodesic		Diameter
SR, LR	[SR,LR]	Mean	Gini		Mean	Gini	
90%, 10%	[20,40]	0.575	0.108	0.566	6.767	0.187	13
75%, 25%	[20,40]	0.562	0.126	0.513	5.323	0.173	8
25%, 75%	[20,40]	0.608	0.120	0.547	4.103	0.185	8

Consistent with the associated increase in edge density, a higher proportion of long-reach nodes led to much smaller average geodesic and diameter in the generated networks (Table 9). Compared to the networks with only 10% long-reach nodes, the networks with 75% long-reach nodes had approximately just over half of the mean geodesic (6.8 to 4.1) and diameter (13 to 8). There was some variation in geodesics between pairs of nodes across all three experiments.

### Varying reach proportions: Three social reaches

Our third set of experiments introduced a third social reach value and varied the proportions of the populations of short-reach (SR), medium-reach (MR) and long-reach (LR) nodes. The social reach values of [30,40,50] are an extension of the longest reaches in the two reaches experiments (at [30,50]). With 75% of shorter reach nodes, those two reach networks had average degree of 31.6 (Table 4), which is similar to the mean degree of 30.7 for the three reach experiments with 70% short-reach population (Table 10). Overall, similar patterns were observed for these three population experiments as for mixing the proportions with two populations.

Consistent with the two reaches experiments where we varied the agent proportions (see Table 7), increasing the proportion of long-reach agents increased the total number of edges are related properties. Unlike the two reach experiments, there was no consistent pattern in Gini coefficient of degree as we varied the mix of nodes (Table 10).

**Table 10:** Varying social reach proportions (3 values): General network properties.

Composition	Reaches	Edges	Density	Degree		Assortativity
SR, MR, LR	[SR,MR,LR]			Mean	Gini	
70%, 20%, 10%	[30, 40, 50]	15,360	0.031	30.7	0.118	0.452
34%, 33%, 33%	[30, 40, 50]	20,553	0.041	41.1	0.162	0.267
10%, 20%, 70%	[30, 40, 50]	30,090	0.060	60.2	0.138	0.232

The mean closeness centrality and mean eigenvector centralities increased as the proportion of longer reach agents increased (Table 11). Gini coefficient for closeness showed no clear pattern whereas the Gini eigenvector decreased substantially from 0.69 to 0.38. These patterns were also found for the ‘Two Circle’ experiments where we varied the agent proportions (Table 8).

The key difference is differences in the property values. This is most evident for the reduction in Gini coefficient of eigenvector centrality, which falls from 0.93 to 0.59 as the longer reach proportion increases in the two reach mixed population and from 0.69 to 0.38 for the three reach experiments. These different ranges can be reconciled by considering the properties from the perspective of number of edges. If the two sets of results (Tables 8 and 11) are combined and ordered by number of edges, the three population centrality measures are simply an extension of the pattern observed for the two population proportion changes.

**Table 11:** Varying social reach proportions (3 values): Centrality measures.

Composition	Reaches	Closeness		Betweenness		Eigenvector	
SR, MR, LR	[SR,MR,LR]	Mean	Gini	Mean	Gini	Mean	Gini
70%, 20%, 10%	[30, 40, 50]	0.231	0.025	0.003	0.625	0.016	0.693
34%, 33%, 33%	[30, 40, 50]	0.273	0.032	0.003	0.602	0.021	0.513
10%, 20%, 70%	[30, 40, 50]	0.308	0.023	0.002	0.393	0.026	0.377

The mean betweenness remained very low across experimental settings (Table 11), with minimal effect from varying the agent mix. Variation in betweenness centrality decreased notably as the proportion of longer reach agents increased.

Clustering coefficient was high across all networks (Table 12). Varying the population mix had minimal effects on this property, and small and inconsistent effects on transitivity and the Gini coefficient of clustering coefficient. This is again consistent with varying the mix of social reaches for two values (Table 9).

**Table 12:** Varying social reach proportions (3 values): Clustering.

Composition	Reaches	Clustering coeff		Transitivity
SR, MR, LR	[SR,MR,LR]	Mean	Gini	
70%, 20%, 10%	[30, 40, 50]	0.580	0.070	0.570
34%, 33%, 33%	[30, 40, 50]	0.591	0.096	0.553
10%, 20%, 70%	[30, 40, 50]	0.600	0.089	0.566

Increasing the proportion of longer reach agents reduced the mean geodesic in networks and the diameter (Table 13). There were no consistent patterns for Gini coefficient of mean geodesic (Table 13).

**Table 13:** Varying social reach proportions (3 values): Geodesics.

Composition	Reaches	Geodesic		Diameter
SR, MR, LR	[SR,MR,LR]	Mean	Gini	
70%, 20%, 10%	[30, 40, 50]	4.333	0.180	8
34%, 33%, 33%	[30, 40, 50]	3.663	0.176	7
10%, 20%, 70%	[30, 40, 50]	3.250	0.179	6

### Property interactions

To use Social Circles to generate networks with specific combinations of properties, users must be able to control property values. We therefore examined not only the impact of algorithm parameters on individual properties but also how the properties are associated with each other.

As the social reach values and proportions are varied, the main properties with patterns are mean degree, assortativity, and mean geodesic. This analysis therefore focusses on the relationship between these properties. We only consider property dependencies for the ‘Two Circles’ experiments with 1,000 nodes; the experiments with 2,000 nodes (results in the Appendix) or three different values of social reach respond to changes in the algorithm parameters with the same patterns as these primary experiments.

**Fig. 3:**
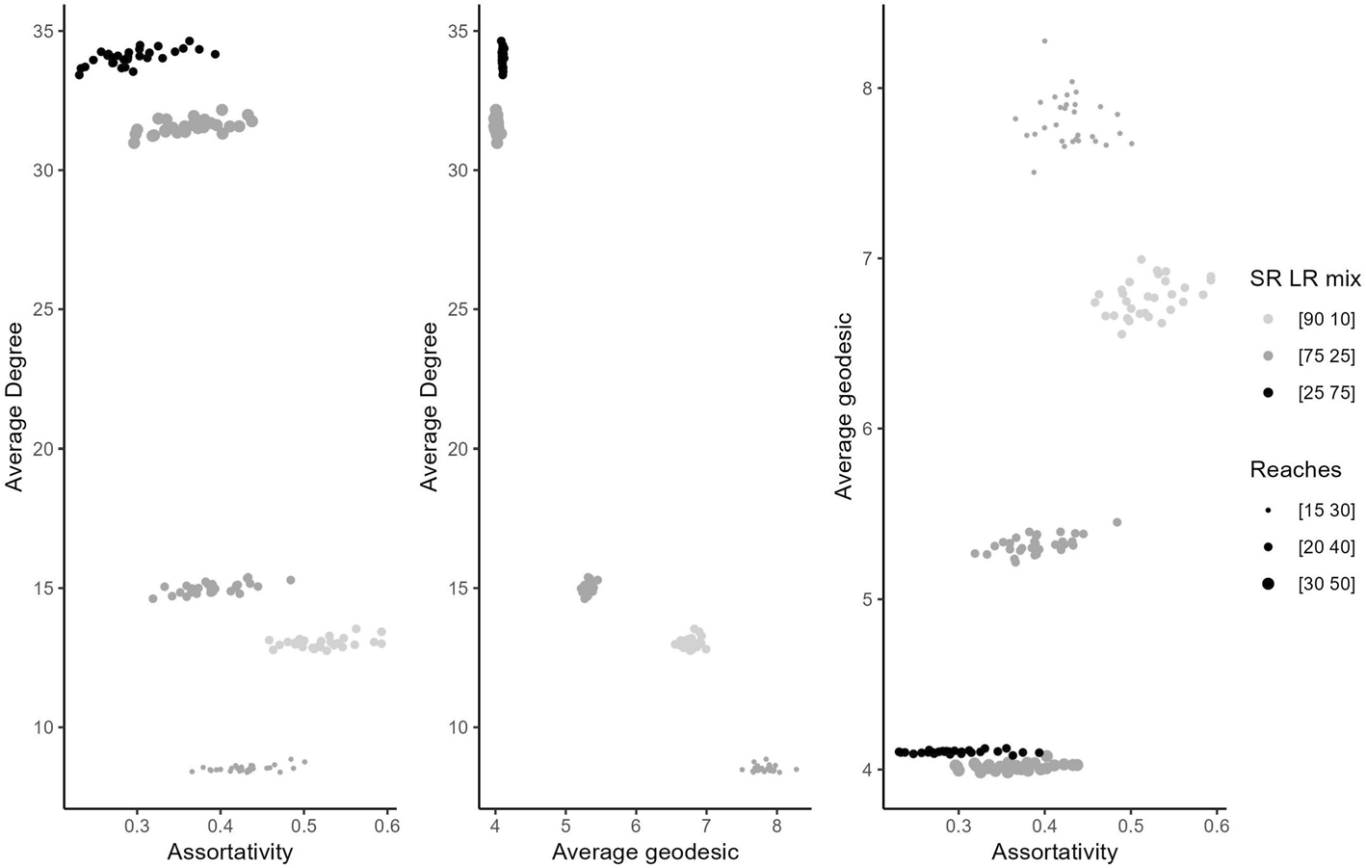
Property interactions for all ‘Two Circles’ experiments, varying the size of social reaches and distribution of social reach values. Average social reach increases as markers become darker (through distribution) or larger (through values).

Figure 3 displays the three properties of interest for both sets of ‘Two Circles’ experiments combined, where we varied the agents’ social reach values and agent proportions. Higher proportions of long-reach nodes (darker points) and longer values of social reach (larger points) are different ways of achieving a larger average social reach. The consequence of both of these manipulations is a higher number of edges or average degree (left and middle panels).

Assortativity decreased as mean degree increased (left panel in Fig. 3) arising from either longer social reaches with fixed proportions or as the proportion of long-reach agents increased with fixed reach values. While assortativity generally decreased as mean degree increased, the relationship is fairly weak. The highest assortativity values are found where 90% of nodes have reach of 20 and 10% have reach of 40, but these networks do not have the lowest mean degree. Further, mean degree is very different across the sets of experiments but assortativity shows only a small change.

We see a similar pattern between the average degree and the network’s average geodesic (central panel in Figure 3) when varying the agents’ social reaches and population mix. As we increase either the agents’ social reach values from (15,30) to (30,50) or increase the proportion of long-reach agents in the population from 10% to 75%, mean geodesic decreases with the increase in average degree. This pattern is stronger than that for assortativity, with large changes in mean geodesic and a consistent decrease as degree increases. However, once average geodesic has fallen to near 4, it does not decrease further with higher degree.

Combining these patterns (right panel of Figure 3) shows that assortativity and average geodesic are directly related in general, an increase (or decrease) in one is associated with an increase (or decrease) in the other. That is, they can only be controlled together through the mediator of average degree. Nevertheless, there is some scope for separate control as the highest values of assortativity (achieved with 90% of nodes with reach of 20 and 10% with reach of 40) have average geodesic values that are quite a bit lower than the networks with longest geodesics.

## Discussion

The mechanism by which the Social Circles algorithm (Hamill and Gilbert 2009) creates networks is spatial and fundamentally depends on the reaches of the nodes. The social reach of a specific node controls the number of opportunities for that node to create edges; a longer reach means more nodes are within the circle and more edges are possible. As expected, where we varied the agents’ social reaches, there were more edges (and higher mean degree and density) with larger social reach values. Similarly, a higher proportion of long-reach agents for any pair of fixed reach values is associated with higher density since it is more likely that a node within the reach of a long reach node will be able to reciprocate and create an edge.

Unless there are systematic structural biases, more edges provide additional paths through the network and therefore shorter geodesics. We found that longer reach values and higher proportions of longer reach nodes are each associated with lower average geodesic, as expected. Average closeness also decreases, as this is simply the node specific average path length to other nodes. There is little variability in node closeness within networks for all sets of networks.

In contrast, mean eigenvector centrality is low but variation is high. Where reaches are short and the proportion of short reach nodes is high, the few long reach nodes that are able to find other long reach nodes have a much higher eigenvector centrality than the majority of the nodes, so variation is extremely high. As either the reaches increase or the proportion of long reach nodes increase, the long reach pairs are able to form edges and locally dense clusters, increasing the proportion of nodes with influential neighbours and evening out eigenvector centrality values.

While the social reach value of a node controls opportunities for edge creation, these edges are only realised if the other node has sufficient reach to reciprocate. This additional mechanism allows separate control of degree distributions and therefore assortativity, which is the correlation of degrees for node pairs defined by edges. Increasing reach values increases the degrees for all nodes (Fig. 1) and is associated with a relatively small decrease in assortativity and variation in degree (Table 4). Increasing the proportion of nodes with higher reach values but fixed reach values increases the degree of longer reach nodes (Fig. 2) and is associated with a larger decrease in assortativity and an increase in degree variation (Table 7). To understand the effect of changing the proportion of longer reach nodes, consider two identical spatial distributions of nodes where some of the shorter reach nodes in for one attempt to create edges have a longer reach in a second attempt. The newly longer reach nodes can reach other nodes that were too far away, increasing their average degree. However, the nodes that remained short reach do not benefit from this change and their average degree remains unaffected. The two consequences of this are a decrease of assortativity and increase in degree variation.

Lastly, there were no particular patterns for clustering coefficient, which is fairly unresponsive to changes in algorithm parameters. This is related to the spatial mechanism of the algorithm, as clustering is ‘determined by the overlap of circles’ (Hamill and Gilbert 2009, paragraph 3.8). If nodes were evenly distributed in space, any nodes that can reach each other to form an edge have at least 39% overlap in their reachable space (Hamill and Gilbert 2009, Box 2), which sets a minimum expected clustering coefficient of 0.39. For all experiments, average clustering coefficient was in the range 0.5 to 0.6.

## Conclusion

This study re-implemented the Social Circles algorithm proposed by Hamill and Gilbert (2009) and extended the experimental design to explore the independent and interactive effects of two key parameters: the values of social reach and the distribution proportions of those reaches. We incorporated a broader range of structural network properties to provide a more comprehensive understanding of how algorithm parameters influence network structure.

There were two main sets of experiments that each involved two reach values. First, we varied the values of the social reaches and fixed the agent proportions. Second, we fixed the reach values and varied the distribution of social reaches in the population. There were two additional sets of experiments, adding a third reach value and increasing the node spatial density. These experiments displayed the same relationships between algorithm parameters and network properties as the main experiments.

Longer reach values and higher proportions of agents with long-reach values were associated with more edges (higher density and mean degree), higher values of closeness and eigenvector centrality, shorter paths (mean geodesics and diameter) and slightly lower values of assortativity. There is limited scope to separately control these properties without also changing mean degree.

There is some opportunity to independently control assortativity through variation in node degrees. Longer reach values increases degree for all nodes, while increasing the proportion of longer reach nodes increases degree only for the nodes with the longer reach.

The two algorithm parameters also have different effects on community concentration in the networks. Communities form around locally dense longer reach nodes. Increasing the proportion of longer reach nodes diffuses these communities and reduces variation in centrality measures.

The spatial nature of the Social Circles algorithm (Hamill and Gilbert 2009) means that clustering coefficient cannot be controlled through algorithm parameters. It is relatively high (between 0.5 to 0.6) for all experiments but similar to property values found in some empirical social networks (Newman and Park 2003).

Overall, Social Circles generates networks with the important features of social networks such as high clustering coefficient, positive degree assortativity and short path lengths. Further, at least some of these properties are directly influenced by the algorithm parameters. However, these properties are tightly associated so the algorithm could not be used to target specific combinations of properties without additional steps.

## Figures and Tables

**Fig. 4:**
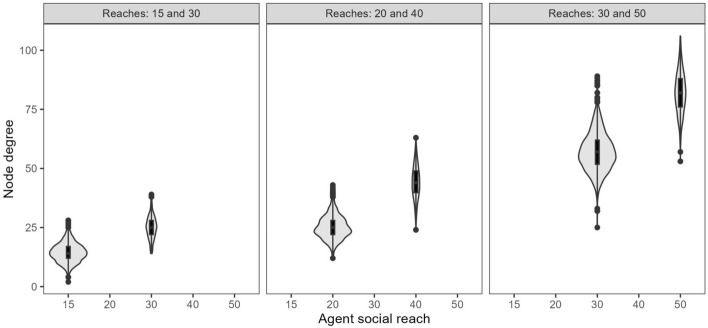
Distribution of node degrees by social reach with varied reach values and fixed proportions of 75% short-reach and 25% long-reach nodes with 2,000 nodes. Longer reaches tend to be associated with higher degree. The pattern is the same as observed for Fig. 1 but with higher degree values.

**Fig. 5:**
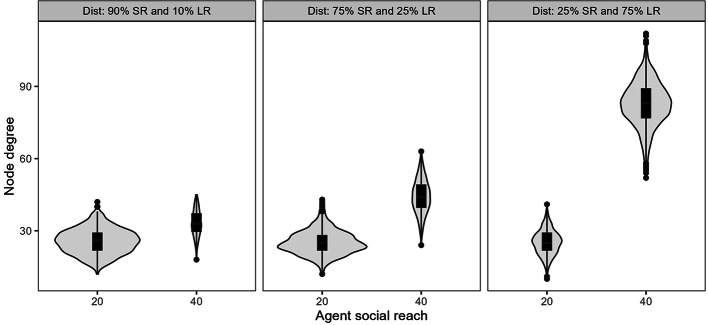
Distribution of node degrees by social reach with fixed reach values and varied proportions of 75% short-reach and 25% long-reach nodes with 2,000 nodes. The pattern is the same as observed for Fig. 2 but with higher degree values.

**Fig. 6:**
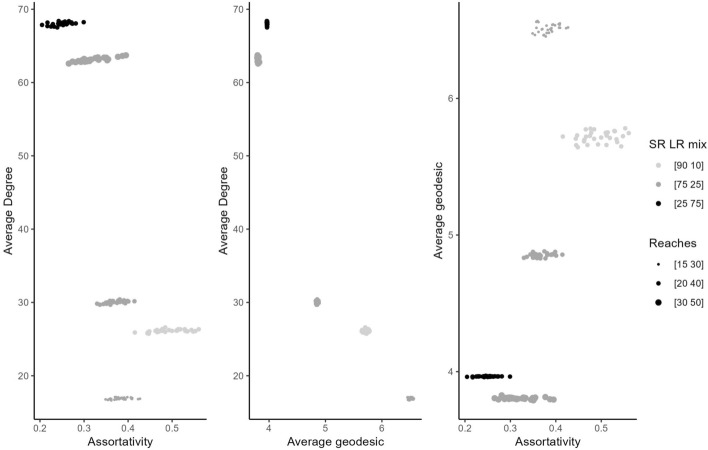
Property interactions for all Two Circle experiments with 2,000 nodes, varying the size of social reaches and distribution of social reach values. Average social reach increases as markers become darker (through distribution) or larger (through values).

## Data Availability

The source code, primary and secondary data used in the model, and documentation can be found on Github under a GNU General Public License 3.0. All data produced by the simulations can be accessed in OSF Repository under a Creative Commons License By Attribution 4.0 International.

## Appendix: 2,000 node results

This appendix includes the additional sets of experiments where we increased the number of nodes in the nominal space to 2,000 and conducted the same sets of experiments varying social reach values and proportion of agents with each value. The experiments with three values of social reach are not provided but are available on request.

The first section of the appendix compares the degree distributions for the nodes with different reach. The second section of the appendix reports the network properties. The final section displays the property interaction plots.

### Degree distributions: density experiments

See Appendix Figs. 4 and 5.

### Network properties with 2,000 nodes

Tables 14, 15, and 16 report the network properties with 2,000 nodes where social reach values vary and the population mix is fixed with 25% assigned the longer reach value. These correspond to Tables 4, 5, and 6 respectively in subsection 3.3.

**Table 14:** Varying social reach values with 2,000 nodes: General network properties.

Composition	Reaches	Edges	Density	Degree		Assortativity
SR, LR	[SR,LR]			Mean	Gini	
75%, 25%	[15,30]	16,881	0.008	16.881	0.197	0.384
75%, 25%	[20,40]	30,038	0.015	30.038	0.178	0.368
75%, 25%	[30,50]	63,116	0.032	63.116	0.117	0.320

**Table 15:** Varying social reach values with 2,000 nodes: Centrality measures.

Composition	Reaches	Closeness		Betweenness		Eigenvector	
SR, LR	[SR,LR]	Mean	Gini	Mean	Gini	Mean	Gini
75%, 25%	[15,30]	0.154	0.031	0.003	0.788	0.006	0.883
75%, 25%	[20,40]	0.207	0.032	0.002	0.775	0.010	0.732
75%, 25%	[30,50]	0.264	0.028	0.001	0.711	0.015	0.501

**Table 16:** Varying social reach value with 2,000 nodes: Clustering and Geodesics.

Composition	Reaches	Clustering Coeff		Transitivity	Geodesic		Diameter
SR, LR	[SR,LR]	Mean	Gini		Mean	Gini	
75%, 25%	[15,30]	0.563	0.121	0.515	6.511	0.175	12.067
75%, 25%	[20,40]	0.564	0.101	0.516	4.852	0.166	8.933
75%, 25%	[30,50]	0.574	0.070	0.550	3.804	0.165	7.000

Tables 17, 18, and 19 report the network properties with 2,000 nodes where social reach values are fixed at 20 for short-reach and 40 for long-reach, and the population mix is varied. These correspond to Tables 7, 8, and 9 respectively in subsection 3.4.

**Table 17:** Varying social reach proportions (2 values) with 2,000 nodes: General network properties.

Composition	Reaches	Edges	Density	Degree		Assortativity
(SR,LR)				Mean	Gini	
90%, 10%	[20,40]	26,136	0.013	26.136	0.120	0.494
75%, 25%	[20,40]	30,038	0.015	30.038	0.178	0.368
25%, 75%	[20,40]	68,029	0.034	68.029	0.201	0.249

**Table 18:** Varying social reach proportions (2 values) with 2,000 nodes: Centrality measures.

Composition	Reaches	Closeness		Betweenness		Eigenvector	
(SR,MR,LR)		Mean	Gini	Mean	Gini	Mean	Gini
90%, 10%	[20,40]	0.175	0.028	0.002	0.783	0.007	0.875
75%, 25%	[20,40]	0.207	0.032	0.002	0.775	0.010	0.732
25%, 75%	[20,40]	0.253	0.028	0.001	0.383	0.016	0.485

**Table 19:** Varying social reach proportions (2 values) with 2,000 nodes: Clustering and Geodesics.

Composition	Reaches	Clustering Coeff		Transitivity	Geodesic		Diameter
(SR,LR)		Mean	Gini		Mean	Gini	
90%, 10%	[20,40]	0.575	0.074	0.567	5.717	0.170	10.233
75%, 25%	[20,40]	0.564	0.101	0.516	4.852	0.166	8.933
25%, 75%	[20,40]	0.609	0.109	0.547	3.965	0.181	8.000

### Property interactions: density experiments

The interactions are the same as observed in Fig. 3 but with higher degree values (Fig.6).
